# From War to Classroom: PTSD and Depression in Formerly Abducted Youth in Uganda

**DOI:** 10.3389/fpsyt.2015.00002

**Published:** 2015-03-03

**Authors:** Nina Winkler, Martina Ruf-Leuschner, Verena Ertl, Anett Pfeiffer, Inga Schalinski, Emilio Ovuga, Frank Neuner, Thomas Elbert

**Affiliations:** ^1^Department of Psychology, University of Konstanz, Konstanz, Germany; ^2^Vivo International (www.vivo.org); ^3^Department of Psychology, University of Bielefeld, Bielefeld, Germany; ^4^Faculty of Medicine, Gulu University, Gulu, Uganda

**Keywords:** child soldiers, war-affected youth, PTSD, war-trauma exposure, post-conflict mental-health support programs

## Abstract

**Background:** Trained local screeners assessed the mental-health status of male and female students in Northern Ugandan schools. The study aimed to disclose potential differences in mental health-related impairment in two groups, former child soldiers (*n* = 354) and other war-affected youth (*n* = 489), as well as to separate factors predicting mental suffering in learners.

**Methods:** Participants were randomly selected. We used the Post-Traumatic Diagnostic Scale to assess symptoms of post-traumatic stress disorder (PTSD) and for potential depression the respective section of the Hopkins Symptom Checklist with a locally validated cut-off.

**Results:** Almost all respondents had been displaced at least once in their life. 30% of girls and 50% of the boys in the study reported past abduction history. Trauma exposure was notably higher in the group of abductees. In former child soldiers, a PTSD rate of 32% was remarkably higher than that for non-abductees (12%). Especially in girls rates of potential depression were double those in the group of former abductees (17%) than in the group of non-abductees (8%). In all groups, trauma exposure increased the risk of developing PTSD. A path-analytic model for developing PTSD and potential depression revealed both previous trauma exposure as well as duration of abduction to have significant influences on trauma-related mental suffering. Findings also suggest that in Northern Ugandan schools trauma spectrum disorders are common among war-affected learners.

**Conclusions:** Therefore, it is suggested the school context should be used to provide mental-health support structures within the education system for war-affected youth at likely risk of developing war-related mental distress.

## Introduction

For at least two decades, civilians in Northern Uganda have been exposed to organized violence including widespread atrocities, child soldiering, and other crimes against humanity. Since 2006, the frequency of violent offenses of the local rebel organization, the “Lord’s Resistance Army (LRA),” has declined, but the memories of war and conflict remain and frequently intrude in the minds of both those who were afraid of being killed and those who were forced to kill, and thus fuel cycles of violence that may even reach the next generations (1). As of 2011, LRA atrocities have shifted to the East of the Democratic Republic of Congo, the Republic of South Sudan, and the Central African Republic where they continue to cause large-scale humanitarian disaster and suffering.

Generally, youth in conflict zones are at risk of developing mental disorders related to their exposure to continuous and traumatic stress. A subgroup of them, namely those associated with armed groups, has been found to be particularly prone to developing trauma-related mental suffering, which includes symptoms summarized under the diagnosis of Post-Traumatic Stress Disorder (PTSD) (2). In line with the Paris Principles (3), we will refer to the Northern Uganda formerly abducted youth interchangeably as child soldiers regardless of abduction duration or duties carried out with the armed group. Self-evidently, those minors’ psychosocial needs appear pressing even after the war has ended, they are freed from captivity, or are related within their families or communities of origin. Beyond the core symptoms of PTSD, survivors in various post-conflict settings have commonly reported high levels of depression and suicidal ideations. So far, however, research has not reached the classroom and investigated education by comparing former child soldiers with youth never associated with armed groups in post-war contexts. Relevant knowledge is mandatory for the efficient set-up of mental-health structures to assist children and youth in post-war periods, particularly in settings in which minors have been forced into child soldiering.

In post-conflict regions, large-scale scholastic support programs are often among the first responses dealing with children of war. This seems essential, especially for child soldiers who suffer substantial disruption of education while with an armed group. On the one hand, for some authors, (re-) placement in educational programs constitutes successful reintegration into social life and they have highlighted the relative benefits of education for war-affected youth, such as socio-economic benefits, peer support, structure of life, and sense of safety (4, 5). On the other hand, PTSD symptoms interfere with scholastic achievement and may ultimately lead to unacceptably high drop-out rates (6, 7). The UN Inter-Agency Standing Committee Guidelines on Mental Health and Psychosocial Support in Emergency Settings therefore suggest a holistic approach to recovery from traumatic experiences, emphasizing both educational and mental-health support (8). That is why we aimed to screen for mental-health disorders in youth already enrolled in educational programs and receiving some level of support.

### Prevalence rates of abduction in northern uganda

Globally, it is estimated that nearly half a million children are involved with armed groups worldwide at any given time. Estimates of abduction incidents by the LRA largely depend on definition. Nonetheless, the occurrence of LRA abductions and their consequences amount to a large-scale humanitarian problem and the necessity for action has not been questioned (9).

With regard to Northern Uganda Vinck et al. (10) found an overall prevalence rate for abduction of 44% in a population-based survey, whereas Annan et al. (11) assessed male youth in Northern Uganda (*N* = 741) and found a third of them reporting histories of abduction. Pham et al. (12) found very similar rates in their population-based survey, namely 33% of Acholi respondents reported having been abducted by the LRA.

### Prevalence rates of PTSD in uganda

A number of studies focusing on PTSD only in the group of formerly abducted youth has been conducted in Uganda. PTSD prevalence rates of 35% were found in a sample of former child soldiers in rehabilitation centers in DRC and Uganda (*N* = 169) (13). It is noteworthy that the majority of studies suggest that approximately every third former child soldier has clinical symptoms of PTSD after release from captivity. These findings have been replicated in the settings of a rehabilitation center (14), and also in a rehabilitation primary school (15) in Northern Uganda. These studies employed highly selective study designs, however, and there was no control group of children who had never been associated with armed groups.

In contrast, one study compared formerly abducted youth recruited in reception centers with selected non-abducted youth in secondary schools and PTSD rates were 27 and 13%, respectively (16). However, the control group in Okello and associates’ study was far from being randomized. Notably, in this study youths interviewed in the reception center did not receive formal education and were not yet integrated into the community; however, both were true for the control group. Two studies conducted in internally displaced people (IDP) camps found significant differences in PTSD rates when comparing the group of child soldiers with other war-affected children (12). Current academic enrollment is not reported in these studies and leaves us only with assumptions about mental-health status and the potential role and benefit of support structures in educational programs. In contrast, the most influential report on Uganda’s youth provided by Annan et al. in cooperation with UNICEF (11) found only mild differences between abductees’ and non-abductees’ emotional distress and social behavior, although it did not assess diagnosis of mental-health disorders such as PTSD. Yet the report adds to the critique of practitioners in the field that a research focus on mental-health-related symptoms in the group of former child soldiers alone might neglect the psychosocial needs experienced by other war-affected children (17). Randomized control group designs have therefore been called for to add crucial information for the enhancement of psychosocial programing for youth in Northern Uganda (17).

### Prevalence rates of depression in uganda

Symptoms of depression in the overall war-affected population of Northern Uganda were reported to be equally high as those of PTSD. Vinck et al. (10) as well as Roberts et al. (18) employed the depression part of the Hopkins Symptom Checklist (HSCL) and reported an overall prevalence rate of potential depression in IDP populations of 45% and 67%, respectively. In comparative studies focusing on the differences between the group of former child soldiers and other war-affected youth in Northern Uganda, repeated significant group differences emerged, with formerly abducted youth revealing more symptoms of depression than their non-abducted peers (16, 19). Formerly abducted youth and non-abducted youth also revealed different rates of current suicidality, with former child-soldiers more frequently reporting current suicidal ideations (16, 20). Findings however continue to suggest a closer look into mediating factors and predictor variables of mental ill-health in both groups.

### Trauma exposure

The majority of studies on formerly abducted and other war-affected youth find convincing evidence that cumulative exposure to trauma stressors predicts the frequency of PTSD. This frequently reported finding, whereby the likelihood for PTSD increases with each traumatic experience, has been called the building block effect (20–24). In Annan et al.’s study (11), high rates of emotional distress were also associated with both committed as well as experienced violence. In line with this, Roberts et al. (18) posited that increased trauma exposure was linked with PTSD and depression in IDP populations in Northern Uganda (18).

Annan et al. (11) additionally found that trauma exposure was the strongest predictor of emotional distress in their sample of Ugandan youth. Also, in various additional studies (12, 18, 25) it was found that exposure to war experiences predicted higher rates of depression and PTSD in Ugandan child soldiers and overall IDP populations. Similarly, Ertl et al. (20) recently found that traumatic events mediated symptoms of depression and PTSD.

Yet Kohrt et al. (26) compared the mental-health status of former child soldiers with that of children never conscripted by armed groups in Nepal and found that former child soldier status was significantly associated with both depression and PTSD outcome measures, and this effect remained significant even after controlling for trauma exposure, suggesting that group differences resulted from additional factors, and not merely from trauma exposure.

### Duration of abduction

The variable of duration of abduction(s) as a mediating factor for developing mental ill-health remains a controversial issue. Up to today authors have failed to find evidence of abduction duration being a significantly associated factor with PTSD or depression symptoms (13, 20, 27). Yet Pham et al. (12) found that respondents who remained in rebel captivity for 6 months and over more frequently met the criteria for symptoms of PTSD and symptoms of depression than those abducted for shorter periods (12). The current survey therefore aimed at understanding the role of duration of abduction as well.

Although there is some evidence regarding state of mind in IDP populations in Northern Uganda, we cannot claim to have valid and congruent research findings with regard to the mental health of former child soldiers compared with non-abducted youth placed within Northern Uganda’s education sector.

Our main research questions therefore were: “Do formerly abducted youth differ in mental health-related impairment from non-abducted war-affected peers in educational settings?” and “What factors predict psychological well-being in war-affected youth in schools in Northern Uganda?”

## Materials and Methods

### Setting

The current study was part of an initial needs assessment of war-affected learners that aimed to enhance the psychosocial care for beneficiaries within existing scholastic support programs carried out by the Windle Trust and by the Norwegian Refugee Council (NRC) in Northern Uganda. The survey built on the network and experiences developed in 2 years of mental-health assessment and referral provision by the international mental-health organization Vivo International (www.vivo.org), who partnered with the above-mentioned organizations and the University of Konstanz in Germany to conduct the current survey.

Ten local trauma counselors who had been trained in basic counseling skills, mental-health diagnosis, and trauma treatment conducted interviews. The interviewers had received 6 weeks of intensive theoretical training and 4 weeks of practical training by a team of clinicians and researchers with degrees from Western universities and extensive work and research experience in East Africa. The screeners learned how to administer a standardized interview for the assessment of PTSD. Prior to the beginning of the present study, local interviewers completed 2 years of working as trauma counselors in Northern Uganda.

Three clinical psychologists (MA or Ph.D.) closely supervised all interviews. The Institutional Review Committee of Gulu University and the Uganda National Council for Science and Technology approved the research protocol.

### Participants

From August 2008 to April 2009, we conducted a school-based survey in Northern Uganda. The survey took place in secondary schools in Gulu, Lira, and Kitgum, as well as in vocational training centers run by NRC in the Gulu and Amuru regions. Youths of these regions had not only experienced war and internal displacement, but were also ethnically and culturally similar, thereby reducing the possibility of political, cultural, or societal biases.

The study population was youth enrolled in scholastic support programs of the above-mentioned organizations. Interviews were carried out in the school compounds in private after a comprehensive explanation of the study was provided and after written informed consent was obtained (signature or fingerprints). There were no personal incentives for taking part in the study.

### Selection procedure

The survey was designed to assess the mental well-being of youth enrolled in formal and informal education support in Northern Ugandan schools. Windle Trust beneficiaries (formerly abducted, orphans, child mothers, or disabled youth) shared public secondary schools with non-supported learners. Therefore, the 12 secondary schools with the largest number of Windle Trust beneficiaries were selected for assessment. Interviewees were selected randomly from lists provided by the partner organization. To provide a comparison group of secondary school learners without organizational support, a class-, age-, and gender-matched comparison interviewee was also enrolled for every beneficiary. Absent learners were contacted and only replaced on the interview lists if they failed to attend a second appointment.

In contrast to the Windle Trust beneficiaries, the NRC beneficiaries went to vocational training centers in which all children received scholastic support who met at least one of the following criteria indicating their vulnerability: orphan, formerly abducted, child mother, and physically handicapped. Therefore, selection procedures varied slightly. From the 10 existing Youth Education Pack (YEP) centers 6 were randomly selected for the survey. These 6 centers provided lists of learners. From the overall learner population, the same proportion of interviewees was randomly selected in each center. In each center more than half of all learners (70 of 120 learners in each center) were interviewed. Absent learners were contacted and only replaced on the interview lists if they failed to attend three appointments.

### Instruments

Local counselors administered clinical interviews with the following instruments.

#### Post-traumatic stress disorder

We used the Post-Traumatic Diagnostic Scale (PDS) (28), which has good psychometric properties and has been used in a wide variety of cultural settings (29–31) e.g., a mental-health assessment of IDPs in Northern Uganda (29–31). Ertl et al. (32) found valid PDS ratings of trained local counselors when comparing the ratings with those of expert clinicians. We established diagnosis of PTSD according to the fulfillment of DSM-IV criteria through the corresponding items in the PDS.

#### Symptoms of depression

Symptoms of depression have most commonly been assessed with the depression section of the Hopkins Symptom Checklist (DHSCL) (33), although its psychometric properties are only moderate. It has been used in samples of refugees in post-conflict countries (32, 34, 35) including Uganda (10, 18), usually selecting a cut-off score of 1.75 to establish a potential episode of major depression. Ertl et al. (32), however, applied DHSCL’s enhanced psychometric properties using a Northern Uganda specific cut-off score for the localized Lou/Acholi version of 2.65. We employed this expert-validated cut-off score for the establishment of a potential diagnosis of an episode of major depression in the current survey, as a lower cut-off score of 1.75 would lead to a large proportion of false positives associated with the high levels of observed general psychosocial distress in the study population and areas.

#### Suicidality

The Mini International Neuropsychiatric Interview (MINI) (36, 37) is a well-established standard diagnostic instrument and has been used in different cultural settings including Uganda (16, 18). We used the suicide section of the MINI as an instrument to assess suicidal ideations and plans in the study sample.

#### Trauma exposure

The Violence, War, and Abductee Exposure Scale (VWAES) is a modified and extended version of the Clinician-Administered PTSD Scale Event Checklist (CAPS) (38), which was specifically designed for formerly abducted and other war-affected individuals in Northern Uganda (32). Given the repeated exposure to violence inherent in the assessed population as well as the difficulties arising when we wanted to assess the number of all individual traumatic events in a lifetime, we relied instead on the number of traumatic event types (e.g., experienced assaults with weapons). Exposure to event types ever was coded only once without encoding frequencies of traumatic events from one event category.

#### Translation

The questionnaire was translated and delivered in Lou, the main language of the Gulu, Amuru, and Kitgum districts. The translation followed recommended guidelines (39), and involved forward and backward translation, and a detailed review by the study team.

### Data analysis

Data were analyzed by using IBM SPSS statistics 19.00. For calculating the path-analytic model we used R 2.10.1. Alpha level was set at 0.05 and two-sided *t*-tests were used to analyze significance. We calculated path analysis to predict PTSD as well as depression scores in war-affected youth in Northern Uganda. Because in the present sample males had been abducted more often than females, we controlled for gender effects by entering residuals corrected for such effects into the model.

Model selection for the path-analytic model was conducted with the AIC criterion. We then used linear regression analysis to evaluate direct and indirect effects on PTSD and depression. Therefore, trauma exposure and duration of abduction were considered antecedent to PTSD. According to the AIC criterion, only trauma exposure was useful for predicting depression.

## Results

### Sample characteristics

Sample characteristics are provided in Table 1. The sample included 355 female (42.1%) and 488 male (57.9%) learners. The mean age of respondents was 19.0 years. The main ethnic group was Acholi, the main religion Christian. Owing to the war, almost all respondents (87.0%, *n* = 733) had been displaced from their home villages at least once in their lives. One out of three female learners reported having been abducted by the LRA at least once in their lives while half of all males reported abduction history. For those who reported histories of abduction, number of abductions varied from one to five times.

**Table 1:** **Sample characteristics of Ugandan war-affected youth respondents *N* = 843**.

Characteristics		Female (*n* = 355)	Male (*n* = 488)
Age in years	Mean (SD)	18.59 (2.60)	19.32 (2.75)
	Median	18.00	19.00
	Range	11–29	10–30
Religion	Christian (%)	99.2	98.0
	Other (%)	0.8	2.0
Ethnicity	Acholi (%)	88.5	88.7
	Other (%)	11.5	11.2
Marital status	Single, never married (%)	88.0	89.8
	Married (%)	1.4	1.0
	Partner/cohabiting (%)	9.9	7.8
	Divorced (%)	7.9	1.4
	Partner died (%)	0.8	0.0
Orphan status	Both parents alive (%)	23.4	25.0
	Maternal orphan (%)	11.9	8.0
	Paternal orphan (%)	33.8	35.5
	Double orphan (%)	31.3	31.6
Ever displaced	No (%)	16.1	10.9
	Yes (%)	83.9	89.1
If ever displaced, how many times?	Mean (SD)	2.60 (4.00)	2.92 (4.74)
	Median	2.00	2.00
	Range	1–40	1–50
Ever abducted?	No (%)	69.3	49.8
	Yes (%)	30.1	50.2
If ever abducted, how many times?	Mean (SD)	1.15 (0.0)	1.20 (0.6)
	Median	1.00	1.00
	Range	1–3	1–5
If ever abducted, for how long (all abductions together in months)?	Mean (SD)	9.35 (16.2)	14.20 (21.5)
	Median	2.00	6.00
	Range	0.001–96	0.01–132

### Exposure to trauma

Cumulative exposure to traumatic stressors as measured by the number of traumatic event types ever experienced was excessive for the group of formerly abducted youth (*n* = 354) with a mean of 18.11 (min = 4, max = 30). In non-abducted youth (*n* = 489), trauma exposure was lower but still considerable with a mean of 9.3 (min = 0, max = 24).

**Figure 1:**
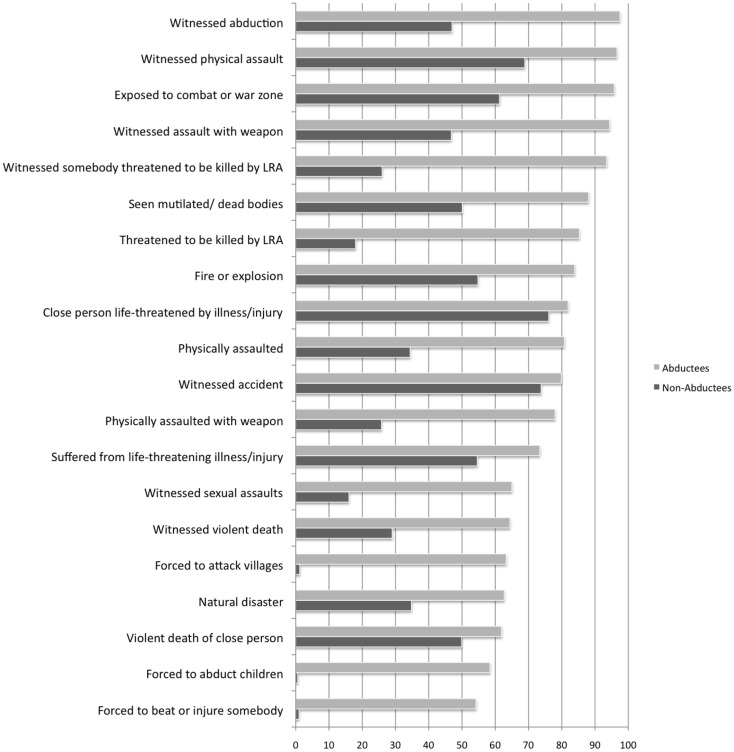
**Most frequent traumatic event types reported by abducted youth compared with non-abducted youth**. Results indicated as percentages.

To compare the means of numbers of traumatic events for the groups of formerly abducted as well as never-abducted youth, a univariate ANOVA was calculated with abduction history and gender as fixed factors. There was a statistically significant main effect of the abduction history variable [*F* (1,835) = 802.60, *p* < 0.001, partial η^2^ = 59], no main effect for the gender variable [*F* (1,835) = 0.001, n.s.], and no interaction effect for both variables [*F* (1,835) = 0.59, n.s.]. The calculations show that formerly abducted youth revealed significantly higher trauma exposure than youth who had never been abducted by the LRA. Most frequent traumatic event types are reported in Figure 1.

### Prevalence of PTSD, depression, and suicidal ideations

**Figure 2:**
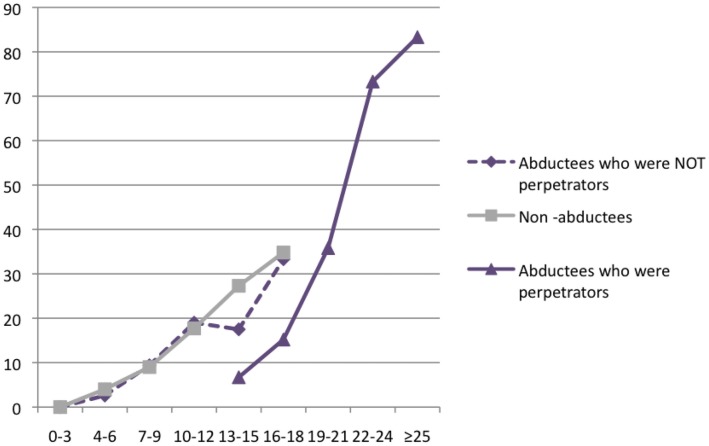
**Prevalence rates of PTSD and trauma exposure in the groups of abducted perpetrators, abducted non-perpetrators, and non-abducted respondents**. Results indicated as percentages.

Almost one-third of abducted youth (32%, *n* = 113) met DSM-IV symptom criteria for PTSD. The PTSD rate for abducted females was 34% (*n* = 37); for abducted males the rate was 31% (*n* = 76). The proportion of non-abducted respondents meeting PTSD criteria was at 12% (*n* = 58) remarkably low and in the lower range for war-exposed groups. Female non-abductees reported a prevalence rate of 16% (*n* = 39), male non-abductees 8% (*n* = 19). Figure 2 illustrates that the prevalence rates of PTSD increase as a function of traumatic event types ever experienced. This finding is true for both abducted and non-abducted learners; however, given the higher levels of trauma exposure in the group of abducted individuals who were forced to harm others, their graph rises higher than that of the non-abducted and abducted non-perpetrator groups. Almost all (87%, *n* = 13) abducted learners who had experienced 25 or more traumatic event types exhibited symptoms of full-blown PTSD. In contrast, the graph for non-abducted youth peaks at a prevalence rate of 35% (*n* = 8) for those who had experienced 16–18 traumatic event types. Hence, as trauma exposure differs between the groups, PTSD prevalence rates also vary.

When the cut-off of 2.65 suggested by Ertl et al. was used for diagnosis of potential depression with the HSCL, 17% of female abductees (*n* = 18) were diagnosed with potential depression as were 7% of male abductees (*n* = 18).

For non-abducted female learners prevalence rate of potential depression was 8% (*n* = 19), for male non-abductees it was 4% (*n* = 9).

39% (*n* = 43) of the female abducted learners reported current suicidal ideations compared with 19% of male formerly abducted respondents (*n* = 47). The rate of suicidality in the group of non-abducted female youth was 29% (*n* = 72), and in male non-abductees it was 16% (*n* = 39).

The χ^2^-tests revealed significant associations between the factors Ever Abducted and Diagnosis of PTSD (χ^2^ = 51.10, *p* < 0.001). No significant associations were found between the variables Ever Abducted and Suicide Risk. For both surveys, χ^2^-tests further revealed that male individuals had more frequently been abducted than female learners (χ^2^ = 32.10, *p* < 0.001).

### Disorders co-morbid to PTSD

30% (*n* = 11) of female formerly abducted respondents meeting symptom criteria for PTSD revealed additional symptoms of depression above the cut-off co-morbid to PTSD. 57% (*n* = 21) of those female formerly abducted youth meeting PTSD criteria reported current suicidal ideations.

17% (*n* = 13) of male formerly abducted respondents meeting symptom criteria for PTSD revealed additional symptoms of depression above the cut-off co-morbid to PTSD.

34% (*n* = 26) of those male formerly abducted youth meeting PTSD criteria reported current suicidal ideations.

### Correlations

Bivariate Pearson correlations were computed for the following variables: PTSD Score, Depression Score, Suicide Risk, Trauma Exposure, and Duration of Abduction.

All clinical measurements, and PTSD Score, Depression Score, and Suicide Risk, revealed strong associations with one another – in both male and female learners. Trauma Exposure was significantly correlated with all clinical measures as well as with Duration of Abduction. Duration of Abduction was notably intercorrelated with PTSD Score as well as with Trauma Exposure, but not with Suicide Risk. Findings are summarized in Table 2.

**Table 2:** **Bivariate correlations between measures**.

	Female respondents (*n* = 355)					Male respondents (*n* = 488)				
	1	2	3	4	5	1	2	3	4	5
1. PTSD score	–	0.55**	0.34**	0.50**	0.10*	–	0.60**	0.37**	0.59**	0.39**
2. Depression score		–	0.53**	0.41**	0.07		–	0.49**	0.44**	0.23*
3. Suicide risk			–	0.34**	0.07			–	0.32**	0.08
4. Trauma exposure				–	0.44**				–	0.49**
5. Duration abductions					–					–

Note. N (female) = 355; N (male) = 488, *p < 0.01, **p < 001 (two-tailed).

### Path-analytic model

Guided by theoretical assumptions and previous empirical research on the effects of cumulative trauma and duration of abduction, we built a path model to uncover potential associations between the variables. Linear regression coefficients were used as path coefficients.

**Figure 3:**
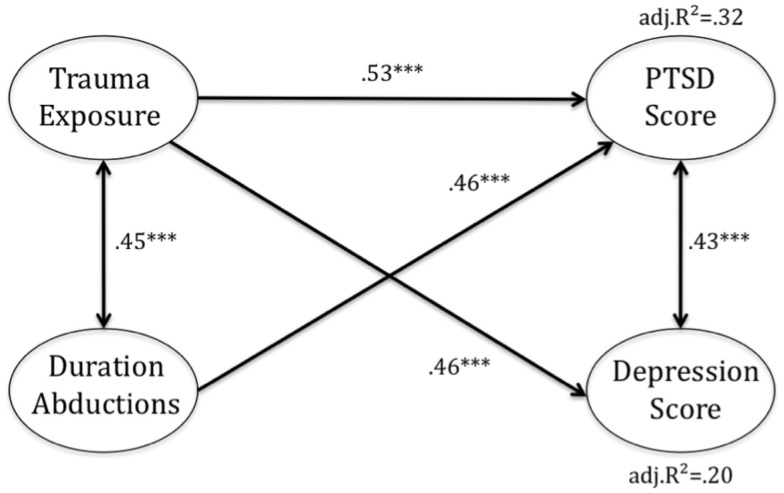
**Path-analytic model for developing post-traumatic stress disorder (PTSD) and depression symptoms after traumatic events and after abduction by the LRA**. The values in the model are standardized linear regression coefficients, correlation, and correlation of residuals. Model selection results from AIC criterion.

Trauma exposure and duration of abduction were modeled as being correlated and having both direct and indirect influence on PTSD and depression. Figure 3 illustrates the results, with standardized linear regression coefficients. Model I indicated two paths to PTSD. Despite a positive association between trauma exposure and abduction duration (*r* = 0.45, *p* < 0.001), trauma exposure and duration of time spent in rebel captivity influenced PTSD severity independently. The linear regression coefficients and respective significance levels are given in Table 3.

**Table 3:** **Summary of simultaneous regression analyses**.

**(A) Summary of simultaneous regression analysis for variables predicting PTSD symptom severity (*N* = 843)**				

**Model 1**	**B**	**SE B**	**ß**	***t***
Constant	0.01	0.19		0.04
Trauma exposure	3.56	0.22	0.53	16.49***
Duration abductions	0.53	0.22	0.08	2.45*
**(B) Summary of simultaneous regression analysis for variables predicting depression score (*N* = 843)**				

**Model 2**	**B**	**SE B**	**ß**	***t***
Constant	0.00	0.02		0.02
Trauma exposure	0.25	0.02	0.45	14.36***

Unstandardized (B) and standardized (ß) regression coefficients. *p < 0.05, ***p < 0.001.

Model II proposes only one direct pathway to depression, namely through trauma exposure. An indirect effect of duration of abduction on depression is illustrated in the path-analytic model, however. The duration of the abduction was indirectly related to the depression score via the correlation with the trauma exposure.

Altogether, the analysis revealed an adjusted *R*^2^ value of 0.32 in Model I and 0.20 in Model II.

Correlations of residuals between PTSD and depression scores were significant.

## Discussion

With the current survey, we aimed to shed more light on factors predicting mental disorders and indexes of restricted functioning in war-affected youth enrolled in school and vocational training programs in Northern Uganda. We sought a better understanding of mental-health issues, comparing two groups, former child soldiers and other war-affected youth, with particular regard to the trauma-related symptoms of PTSD and depression. Research was carried out as a prerequisite to enhancing psychosocial service provision for war-affected youth in Northern Uganda.

In a gender-balanced sample of highly war-affected youth with almost all respondents reporting experiences of displacement, we found 69.3% of all interviewed male youth and 49.8% of females reported abduction history. In line with current research in Uganda and elsewhere we found higher levels of PTSD and potential depression in former child soldiers than in war-affected youth never conscripted into the LRA. More female learners were diagnosed with potential depression in both groups than male learners. Current suicidality was higher in female former child soldiers than in female non-abducted learners; the same was true for male abductees versus non-abductees. Yet rates of suicidality were generally higher for females than for males.

This observation needs to be kept in mind for programing. We found significant group differences of trauma exposure between the groups of abductees and non-abductees and a building block mechanism of trauma exposure, which was valid for both groups. In a path-analytic model, the extent of exposure to traumatic stressors proved itself to be a predictor for both PTSD and depression scores, having direct effects on both outcome variables. Yet we suggest that an additional factor beyond trauma exposure is important in predicting mental illness in Northern Ugandan war-affected youth. A variable combining the information of abduction history with duration of abduction and coded zero for all non-abductees was found to be of additional relevance in the path-analytic model predicting PTSD outcome score via an independent path as well as having an indirect effect on depression and PTSD score via trauma exposure.

Limitations of the study concern the selection of the sample from children enrolled in school. Thus findings cannot be generalized to the overall population of war-affected youth in Uganda. They surely indicate, however, that psychological suffering is not only common in IDP camps (2, 20) but similarly common in the education sector in Northern Uganda. Taking into account the large numbers of youth with displacement and abduction history in the area, the results draw attention to a large-scale problem inherent in Northern Ugandan schools.

Interviews were conducted with the help of trained local interviewers; however, a validation study with expert clinicians using the same questionnaire design has been used and discussed elsewhere (32).

We observed group differences between former child soldiers and other children who were not recruited by the rebels but were still affected by the violent conflicts in rates of PTSD and depression. Yet it needs to be taken into account that in the group of war-affected but never conscripted youth who had experienced 16–18 traumatic event types the PTSD prevalence rate was also notably high at 34.8%. Youth of both groups with PTSD diagnosis often revealed co-morbid symptoms of depression and suicidal ideations underpinning their mental suffering. When looking more closely at potential risk factors for developing PTSD, we found extreme levels of trauma exposure in the group of child soldiers with a maximum of 30 different traumatic event types experienced, significantly discriminating the groups of concern. Interestingly, we found the same mechanism, namely the building block, in all violence-exposed groups of respondents, obtaining almost parallel graphs with the same peak for those who did not commit perpetrator events. In contrast, the building block effect was delayed for those abductees who had committed perpetrator events. This finding suggests that prevalence rates of PTSD increase as a function of trauma exposure in both groups. Yet it also suggests that possibly the trauma network functions differently for those who were not only victims but also perpetrators. It is possible that owing to training and combat experience their perception of potentially traumatic situations and helplessness during those events varies from that of non-combatants. Nonetheless, from this finding it appears that the resilience of every individual can be shattered once a certain individual threshold of trauma exposure has been reached. Consequently, trauma exposure must be taken into account when we screen children with the highest risk of mental-health disorder. In future, event scales could help to develop more feasible screening procedures, being especially suitable for lay staff. At the same time the building block illustrates that every youth with past traumatic events has an increased vulnerability to PTSD; the more event types, the higher their vulnerability. Needless to say, the prevention of further war, domestic, and/or gender-based violence is therefore of outmost relevance in the prevention of psychological ill-health in war-affected youth *with* or *without* abduction history.

Suicidal ideations in learners were more commonly reported in former child soldiers than in non-abductees and were more frequent in females than in males. In girls, we found that two out of five former abductees and every fifth non-abducted exhibited suicidal thoughts or plans within 4 weeks prior to the interview.

This finding leads us to suggest that both groups of affected youth should benefit from any kind of psychosocial support in post-war contexts. Crisis intervention strategies and referral for emergency client cases seem to be of greatest relevance for both groups of learners. The need to roll out service provision by lay counselors to the school environment appears to warrant urgent attention. Child Disarmament, Demobilization and Reintegration (DDR) programs and other support programs would be well advised not to exclude specific groups of youth from psychosocial program support, but to implement community youth programs with a focus on treating mental-health disorders and violence prevention on a large scale.

In line with previous research studies, we replicated the findings that, first, trauma exposure discriminated the groups of child soldiers and non-abductees and, second, that increased trauma exposure did increase the likelihood of developing PTSD in the groups of concern. Also, in the path-analytic models trauma exposure predicted both PTSD symptoms and symptoms of depression, and hence it was partly responsible for the different levels of mental-health diagnosis found in both groups. In Kohrt’s (26) study group differences also remained stable when they controlled for the variable of trauma exposure. Therefore, the variable of abduction duration, which also entailed abduction history, was of additional relevance in the path-analytic model. The variable explained part of the variance of the PTSD score via an independent path. Hence, those who were abducted longer had more symptoms of PTSD independent of trauma exposure. An indirect influence of duration abduction via trauma exposure was also found to be significant in the models predicting PTSD as well as depression scores, however. In contrast, abduction duration did not independently predict symptoms of depression. Consequently, all learners abducted or not, with high trauma exposure are at risk of developing symptoms of PTSD and depression. This effect is aggravated by the duration of abduction in former child soldiers. Child soldiering and the duration of abduction, however, predicted PTSD scores via an independent path. We can only hypothesize about the processes behind this finding, but it seems feasible that a great sense of helplessness and fear experienced throughout the abduction without experience of various distinguishable traumatic event types during the abduction could lead to PTSD directly and affect the resilience of individuals more strongly. Longer duration of abduction also leads to a longer interruption of age-adequate development including scholastic and social development and interruption of family ties. This probably has an effect on how child soldiers perceive themselves during and/or after abduction as well as how they are perceived by others after their relocation back in their communities, possibly leading to more frequent symptoms of depression.

The results reported here indicate the need to put psychological support structures in place, and this applies also to learners already enrolled in scholastic support programs or formal education. Comparable rates of full-blown PTSD in IDP settings and education settings imply that schools can serve as low threshold programs and absorb learners who have experienced frequent war trauma and exhibit persistent symptoms of PTSD and depression. At the same time and in line with the UNICEF (3) report cited earlier the findings equally emphasize that “education & vocational training are by no means a cure-all” for war-affected youth in Northern Uganda. Yet the Ugandan schools’ potential to serve as an entry-point for case management and further mental-health programing can be acknowledged. We also agree that structured days, ongoing learning opportunities and social support provided by teachers and peers in schools are meaningful resources for youth integration and participation as well as the development of future-oriented positive attitudes. Mental-health diagnosis, however, always implies that impairment in all-day functioning often interferes with school performance or leads to increased drop-out rates. We suggest that the provision of psychosocial care in schools will mitigate impairment, poor school performance, and increased school drop-out rates associated with mental illness. The numbers of learners with diagnosis of PTSD and/or potential depression suggest the need for embedded provision of psychological support in schools and vocational training centers in Northern Uganda. Case management systems seem essential for those revealing co-morbid disorders. Such support must address symptoms of PTSD and depression; the following implementation logic seems not only plausible, but also feasible. First, psycho-education as well as explanations about war experiences and normalization of symptoms could be mainstreamed into the school curriculum to insure accessibility of all learners and provide them with general coping mechanisms. Second, teachers could be trained to detect as well as generally deal with mental health-related symptoms in the classroom and offer referral pathways for those in need of individualized treatment. Third, psychological treatment components could be delivered within the schools with adequate teacher training. All three approaches have already been explored in Northern Ugandan schools run by the above-mentioned partner organizations and are having considerable success in terms of the reintegration, recovery, and reconciliation of the affected learners.

## Conclusion

Mental-health intervention strategies with a focus on trauma-related symptoms including those of PTSD, depression, and suicidal ideation are needed to assist survivors in reducing their burden of mental suffering and to improve their performance in school. Impaired functioning was very frequently related to having experienced these stressors; therefore ways on how to cope with ongoing stress also appear to be essential in psychosocial programing. As long as the mental suffering of youth in post-war contexts and equally their human right to treatment and care are not fully acknowledged by the world community, attempts to enhance support structures will remain limited. Through the lens of the ongoing LRA violence in the Democratic Republic of Congo, the Republic of South Sudan, and the Central African Republic future needs to treat their suffering youth are inevitable.

## Author Contributions

NW made substantial contributions to the conception and design, supervised the data collection, analyzed and interpreted data, and drafted the manuscript. MR-L made substantial contributions to the conception and design, collected data, made substantial contributions to the interpretation of data, and revised the manuscript. VE collected data and revised the manuscript. AP collected data and revised the manuscript. IS made substantial contributions in data analysis and revised the manuscript. EO made contributions to the conception of study, interpretation of data, and made substantial contributions to revision of the manuscript. FN made substantial contributions to the conception and design, interpretation of data, and manuscript revision. TE made substantial contributions to the conception and design, interpretation of data, and manuscript revision. All authors read and approved the final manuscript.

## Conflict of Interest Statement

The authors declare that the research was conducted in the absence of any commercial or financial relationships that could be construed as a potential conflict of interest.
